# Cognitive decline and reduced bone mineral density under the bone-brain axis: mechanistic insights and imaging evaluation strategies

**DOI:** 10.3389/fnagi.2026.1799025

**Published:** 2026-05-20

**Authors:** Yunhai Mao, Zhe Sui, Mengchao Zhang, Yihang Ma

**Affiliations:** 1 Department of Radiology, China-Japan Union Hospital of Jilin University, Changchun, China; 2 Department of Spine Surgery, China-Japan Union Hospital of Jilin University, Changchun, China

**Keywords:** Alzheimer’s disease (AD), bone-brain axis, cognitive decline, multimodal imaging, osteoporosis

## Abstract

Against the backdrop of an accelerating global aging population, the epidemiological correlation between cognitive impairment and osteoporosis has become increasingly prominent. These two conditions exhibit a profound pathological coupling mediated by the bidirectional regulatory network of the “bone-brain axis.” The operation of this axis is rooted in an intricate neuro-skeletal signaling network involving hormonal dysregulation, systemic inflammatory cascades, and the aberrant regulation of core molecular pathways, such as Wnt/β-catenin and RANKL/OPG. Together, these factors synergistically drive the synchronized pathological progression of enhanced bone resorption and neurodegeneration. To address these complex pathological interactions, clinical evaluation strategies are undergoing a paradigm shift, transitioning from single-modality assessments toward the deep integration of multimodal imaging. By fusing cutting-edge technologies—including structural/functional MRI, molecular PET imaging (targeting Aβ and Tau deposition), and high-resolution peripheral quantitative computed tomography (HR-pQCT)—researchers can now comprehensively characterize the spatiotemporal patterns of bone microstructural degradation and brain functional evolution across scales ranging from the microscopic to the macroscopic. Prospectively, leveraging deep learning algorithms such as 3D-CNN to integrate multimodal biomarkers and construct risk-prediction models for bone-brain comorbidities will emerge as a pivotal pathway for achieving early precision screening and personalized preventive intervention for Alzheimer’s disease (AD).

## Introduction

Against the global backdrop of an accelerating aging population, cognitive impairment and osteoporosis have emerged as prevalent conditions severely threatening the physical and mental wellbeing of the elderly, garnering extensive attention from both academia and society (Gerosa and Lombardi, 2021). A growing body of evidence indicates that cognitive decline and osteoporosis are frequently correlated and comorbid in terms of both epidemiology and pathogenic mechanisms (Kostev et al., 2018; Kwon et al., 2021; Loskutova et al., 2019; Zhang et al., 2023). Within this context, the emerging field of the “bone-brain axis” has become a subject of sustained interest and intensive exploration.

The neuro-skeletal crosstalk involves intricate signaling networks, including hormonal regulation, inflammatory pathways, and the bidirectional modulation of key molecular pathways such as Wnt/β-catenin, RANKL/OPG (Glasnović et al., 2020; Liu et al., 2024). These findings not only reveal a profound pathological coupling between the bone and the brain but also provide a theoretical foundation for developing integrated intervention strategies—such as anti-inflammatory therapies and hormone replacement treatments—targeting the bone-brain axis.

However, imaging research investigating the link between osteoporosis and brain structure/function remains in its infancy. Effectively integrating bone density data with cranial radiological features to construct cross-modal prediction models remains a critical bottleneck and a core challenge for achieving early risk assessment of Alzheimer’s disease (AD) and osteoporosis comorbidity.

This article systematically reviews the clinical correlations, molecular mechanisms, and imaging features of cognitive decline and osteoporosis, while discussing the diagnostic value and limitations of imaging technologies within the AD continuum and osteoporosis. Emphasis is placed on the pivotal role of the “bone-brain axis” in the occurrence of comorbidities. Based on current research, we propose an integrated screening framework combining bone density and multimodal imaging of AD, aiming to provide interdisciplinary diagnostic insights for future research.

## 2 Clinical features and diagnostic framework of Alzheimer’s disease and osteoporosis

### 2.1 Alzheimer’s disease continuum

Cognitive decline associated with Alzheimer’s disease (AD) is a prolonged and continuous process, with the clinical spectrum spanning from Cognitively Unimpaired (CU) and Mild Cognitive Impairment (MCI) to severe dementia (Jack et al., 2018; Scheltens et al., 2021). Modern clinical diagnostic models have transitioned from a reliance on purely symptomatic staging toward a biological diagnostic system centered on the AT(N) framework (Amyloid/Tau/Neurodegeneration) (Jack et al., 2024; Lu et al., 2024; VandeVrede et al., 2025).

- Core biomarkers: Diagnosis relies on cerebrospinal fluid (CSF) testing or molecular PET imaging to confirm the presence of β-amyloid (Aβ) pathology (Core Type 1 Biomarker) and tau pathology (Core Type 2 Biomarker).
- Neurodegeneration (N): Evaluating brain volume atrophy (such as hippocampal atrophy) via structural magnetic resonance imaging (MRI) allows for the assessment of cognitive impairment severity at a macroscopic level.

### 2.2 Overview of osteoporosis characteristics

Often referred to as a “silent disease,” osteoporosis is a chronic skeletal condition characterized by reduced bone mass, deterioration of bone microarchitecture, and compromised bone strength. Due to the absence of overt early symptoms and its insidious progression, the condition frequently remains undiagnosed until a fragility fracture occurs (Ayers et al., 2023; Rachner et al., 2011). Patients with osteoporosis face a lifetime fracture risk as high as 40%, with fractures most commonly occurring in the spine, hip, or wrist (Vilaca et al., 2022). Based on its etiology, osteoporosis is classified into two primary categories: primary and secondary (Amarnath et al., 2023).

## 3 Synchronous bone-brain degeneration: the bone-brain axis

Clinical research indicates that skeletal health is generally poor in patients with dementia, who also exhibit a higher susceptibility to fractures (Loskutova et al., 2009; Wang et al., 2014). Specifically, patients in the early stages of AD demonstrate lower bone mineral density (BMD), a metric that significantly correlates with both brain volume and memory performance (Kalc et al., 2023); conversely, AD patients with higher BMD scores exhibit superior memory retention.

Findings from cohort studies further demonstrate that low femoral neck BMD in women is associated with an increased risk of cognitive decline (Tan et al., 2005). Subsequent research has confirmed that the incidence of cognitive impairment is notably higher among patients with osteoporosis (Lin et al., 2021; Zhao et al., 2023). Furthermore, higher BMD has been linked to a reduced burden of white matter hyperintensities (WMH) and superior performance in specific cognitive domains—an association that remains independent of established vascular risk factors (Stefanidou et al., 2021).

These findings underscore a significant bidirectional correlation between cognitive decline and skeletal disorders. Mechanistic studies reveal that the central nervous system (CNS) can directly regulate the skeleton through efferent nerves. Moreover, various central neurohormones (e.g., follicle-stimulating hormone), neuropeptides (e.g., neuropeptide Y), and neurotransmitters (e.g., glutamate), along with their intracellular signaling pathways, are actively involved in the modulation of bone metabolism (Herber et al., 2019; Huang et al., 2019; Zhang et al., 2023).

**FIGURE 1:**
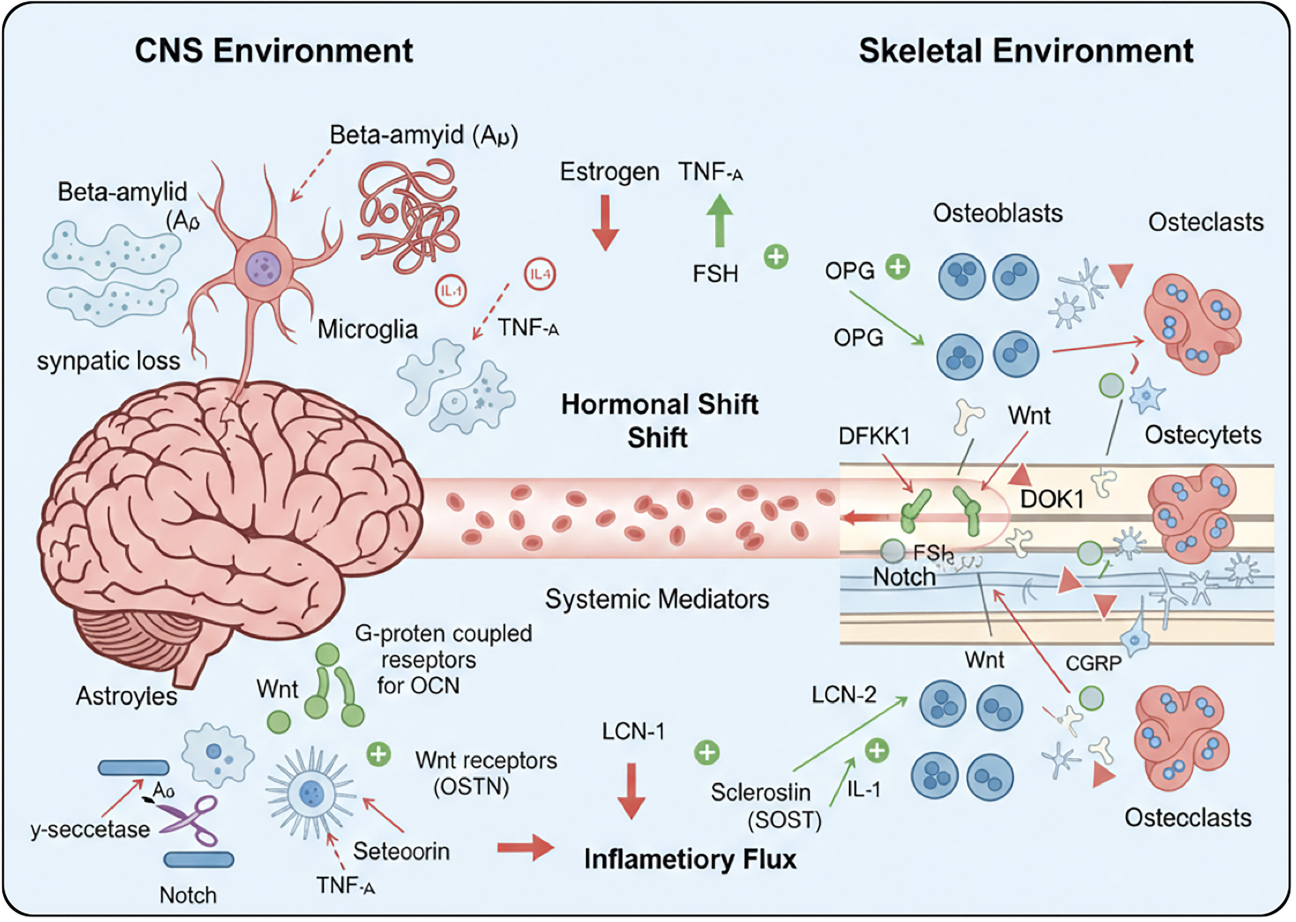
Schematic representation of bidirectional bone-brain interactions and their pathophysiological mechanisms.

In addition to its traditional roles in support, protection, hematopoiesis, and mineral storage, bone also serves as a critical endocrine organ. It is capable of perceiving and integrating diverse stimuli to transmit signals to other tissues, making it a pivotal regulatory organ (Gerosa and Lombardi, 2021; Liang et al., 2024; Obri et al., 2018; Yu et al., 2025; Zhou et al., 2021). In summary, the brain and bone tissue interact through the “bone-brain axis” via neural, humoral and other pathways thereby synergistically influencing the onset and progression of associated diseases (Figure 1).

### 3.1 Hormonal modulation

Within the precise and complex physiological system, hormones act as refined regulatory hubs, playing a pivotal role in modulating both cerebral cognitive function and skeletal health.

#### 3.1.1 Neurohormones

Neurohormonal regulation of the bone-brain axis is primarily realized through the hypothalamus-pituitary-peripheral circulation axis, with the most representative being the hypothalamic-pituitary-gonadal (HPG) axis and the hypothalamic-pituitary-thyroid (HPT) axis (Xiong et al., 2022). In this precise hierarchical regulatory system, the hypothalamus serves as the central integration site, secreting releasing hormones to stimulate the pituitary to synthesize and secrete tropic hormones (such as follicle-stimulating hormone, FSH; and thyroid-stimulating hormone, TSH), which in turn regulate peripheral effector hormones and directly participate in bone metabolism (Rusch et al., 2023).

Epidemiological evidence indicates that peri- and postmenopausal women often simultaneously present with cognitive decline and osteoporosis (Ettleson et al., 2024; Guo et al., 2025; Kistler-Fischbacher et al., 2021; Reeves et al., 2021; Sowers et al., 2003). This stage is typically characterized by elevated levels of FSH accompanied by a decline in estrogen levels. Recent research has confirmed that, besides its involvement in menopause-related bone loss, pathological abnormalities of the HPG axis also play a critical role in Alzheimer’s disease (AD). Elevated FSH can cross the blood-brain barrier (BBB) and bind to FSH receptors (FSHR) expressed on hippocampal and cortical neurons (Xiong et al., 2022). This binding activates the C/EBPβ-δ-secretase signaling pathway, directly accelerating the cleavage of amyloid precursor protein (APP) and the phosphorylation of Tau protein, thereby driving the core pathological processes of AD independently of estrogen levels. Research has demonstrated that estrogen is paramount for maintaining normal brain function. On one hand, it facilitates the degradation of APP and modulates processes related to oxidative damage and energy metabolism (Li et al., 2021; Luo et al., 2022); on the other hand, estrogen exerts neuroprotective effects by alleviating β-amyloid (Aβ)-induced lipid peroxidation and glutamate-mediated excitotoxicity (Sochocka et al., 2023). Furthermore, its neuroprotective role is reflected across multiple dimensions, including the maintenance of volume in cognitive-related brain regions and the regulation of synaptic function (Bagit et al., 2021; Brann et al., 2022). Consequently, a physiological decline or pathological deficiency in estrogen levels is linked to an increased risk of cognitive impairment in the population. Similarly, estrogen maintains bone homeostasis by promoting the differentiation of mesenchymal stem cells into osteoblasts and inducing osteoclast apoptosis, thereby exerting an anti-osteoporotic effect (Boyle et al., 2021). The precipitous drop in estrogen levels post-menopause leads to an imbalance in bone resorption. Beyond directly affecting bone remodeling, declining estrogen levels also trigger an increase in various inflammatory cytokines (such as Interleukin-1) and alter the distribution of immune cells, ultimately compromising skeletal stability (Cheng et al., 2022; Fischer and Haffner-Luntzer, 2022).

Thyroid-stimulating hormone (TSH) is a glycoprotein hormone regulated by the hypothalamic-pituitary-thyroid (HPT) axis. As a critical systemic messenger, it possesses dual roles: it regulates thyroid function and participates in the modulation of cognition and bone remodeling. Regarding pathological abnormalities related to Alzheimer’s disease (AD), clinical evidence indicates that reduced TSH levels are often consistent with the severity of cognitive decline. This reduction in TSH is increasingly recognized because of AD-related neurodegeneration: β-amyloid (Aβ) deposition and Tau pathology can trigger pituitary atrophy or hypothalamic dysfunction, thereby disrupting the feedback loop of the HPT axis (Marouli et al., 2021; Salehipour et al., 2023). In the skeletal microenvironment, TSH can bind to TSH receptors (TSHR) expressed on the surface of osteoclast precursors and mature osteoclasts, exerting a potent and direct inhibitory effect on bone resorption. Additionally, TSHR signaling in osteoblasts can promote the production of osteoprotegerin (OPG), further shifting the RANKL/OPG balance toward bone protection. Consequently, the HPT axis constitutes an important neuroendocrine circuit; its functional abnormalities in AD can synergistically accelerate neurocognitive decline and osteoporotic bone loss (Zaidi et al., 2023).

#### 3.1.2 Bone-derived hormones

Osteocalcin (OCN) is a non-collagenous protein secreted by osteoblasts and encoded by the *Bglap* gene. As a 5 kDa protein comprising 49 amino acids, it is an indispensable marker of bone formation during the mineralization process (Karsenty, 2023; Ruggiero et al., 2024). OCN exists in two forms: carboxylated (cOCN) and uncarboxylated (ucOCN). While cOCN primarily accumulates within the bone matrix, ucOCN is released into the systemic circulation and exerts regulatory effects on multiple organs (Bu et al., 2025; Razny et al., 2017; Tacey et al., 2020).

Upon entering the bloodstream, ucOCN can cross the blood-brain barrier (BBB) via passive diffusion or through osteocyte-derived extracellular vesicles (EVs) to enter the central nervous system. This EV-based transport mode is crucial for maintaining cerebral OCN levels; however, its transport efficiency declines significantly with age, which may serve as a pivotal pathological mechanism for central OCN deficiency in elderly individuals and patients with Alzheimer’s disease (AD). Once inside the central nervous system, OCN is widely distributed across key brain regions—including the hippocampus (encompassing CA1, CA3, and the dentate gyrus), the amygdala, and the brainstem—and primarily acts on neurons. At the receptor level, OCN specifically binds to Gpr158 and GPR37 receptors on the neuronal surface, activating downstream signaling pathways to modulate the synthesis and release of neurotransmitters such as serotonin, dopamine, and GABA, thereby enhancing cognitive and memory functions (Bian et al., 2024; Oury et al., 2013). Concurrently, at the glial cell level, OCN optimizes the cerebral energy metabolism environment by upregulating glycolysis in astrocytes and microglia, which subsequently enhances the microglial uptake and degradation of Aβ, effectively alleviating the AD-related amyloid plaque pathological burden (Jiang et al., 2022; Shan et al., 2023).

Lipocalin-2 (LCN-2) is primarily secreted by osteoblasts, and its expression level in bone tissue is more than 10 times higher than in other tissues (Zeng et al., 2022). Research has shown that LCN-2 is significantly upregulated during the process of osteoblast differentiation and is closely related to skeletal development and bone turnover (Jaberi et al., 2021). As a secreted protein with a relatively small molecular weight, LCN-2 can directly cross the blood-brain barrier (BBB) from the systemic circulation and enter the central nervous system. It acts on the paraventricular nucleus (PVN) of the hypothalamus, where it functions to suppress appetite and regulate metabolism by binding to and activating the melanocortin 4 receptor (MC4R) signaling pathway. At the molecular receptor level, the biological effects of LCN-2 are primarily mediated by specific surface receptors 24p3R and Megalin. Notably, plasma LCN-2 levels are significantly elevated as early as the preclinical stage of Alzheimer’s disease (AD) and are closely correlated with Aβ levels in the cerebrospinal fluid; this suggests that this hormone and its receptor pathway may play a key targeted regulatory role in the early pathological evolution and neuroinflammatory triggering of AD (Eruysal et al., 2019).

Osteocrin (OSTN, also known as Musclin) is a bone-derived protein (Ataman et al., 2016). Within skeletal tissues, OSTN is primarily expressed in osteoblasts and osteocytes, particularly reaching high expression levels during early bone formation to promote growth; however, its levels gradually decline with age (Thomas et al., 2003). In the developing cerebral cortex, OSTN serves as a regulatory factor for dendritic growth in response to sensory experience. Recent studies have demonstrated that OSTN can prevent depression-like behavior in female mice by activating urocortin 2 signaling pathways in the hypothalamus and improving inflammatory biomarker expression in monocytes, suggesting a protective role against neuroinflammation (Cho et al., 2024).

Sclerostin (SOST) is a protein expressed by mature osteocytes. It binds to low-density lipoprotein receptor-related proteins on the surface of osteoblasts, blocking the Wnt signaling pathway and thereby inhibiting the proliferation and differentiation of osteoblasts (Delgado-Calle et al., 2017; Gao et al., 2023). Research has confirmed that SOST in the systemic circulation can cross the blood-brain barrier (BBB) and enter the brain parenchyma through mechanisms such as passive transport. Upon entering the central nervous system, the primary target of SOST is low-density lipoprotein receptor-related protein 5/6 (LRP5/6); by binding to these receptors, SOST can competitively inhibit the canonical Wnt/β-catenin signaling pathway. In the pathological context of Alzheimer’s disease (AD), this mechanism is closely related to the deposition of cerebral β-amyloid (Aβ): clinical data indicate that elevated plasma SOST levels in the elderly population are significantly correlated with an increased brain Aβ load. Furthermore, because serum SOST levels have a clear correlation with bone mineral density (BMD), this further elucidates how SOST acts as a key secreted molecule, linking peripheral skeletal metabolic status to degenerative changes in the central nervous system (Chauhan et al., 2023; Shi et al., 2024; Yuan et al., 2023).

### 3.2 Inflammatory pathways

Inflammation plays an indispensable and critical role within both the nervous and skeletal systems, with a dense and complex intrinsic link existing between these two major systems. Upon receiving specific stimuli, glial cells in the central nervous system (CNS)—including astrocytes and microglia—rapidly release a series of pro-inflammatory cytokines, such as interleukin-1 (IL-1) and tumor necrosis factor-α (TNF-α) (Kinney et al., 2018). These cytokines act as “disruptors” that not only directly compromise the normal physiological functions of neurons—leading to neuronal injury or even death—but also promote the deposition of β-amyloid (Aβ), the formation of phosphorylated tau protein (P-tau), synaptic loss, and neurodegeneration. Furthermore, through an array of complex signaling pathways, they interfere with the normal transmission of neurotransmitters, thereby exerting a severe impact on overall neurological function (Deng et al., 2024; Xie et al., 2023). Crucially, once these inflammatory cytokines enter the systemic circulation, they trigger a systemic inflammatory response. Acting as a “catalyst,” they promote the formation of osteoclasts and enhance their bone-resorption capacity. Concurrently, they inhibit the normal function of osteoblasts, obstructing the bone-formation process and ultimately leading to bone loss, osteoporosis, and other pathological conditions (Iantomasi et al., 2023). The secretion processes of certain neurotransmitters and neuropeptides are also disturbed by inflammation, and these substances can act directly on skeletal cells to modulate bone metabolism. For example, the secretion levels of neuropeptides such as calcitonin gene-related peptide (CGRP) change significantly under inflammatory conditions, which in turn affects the activity of both osteoblasts and osteoclasts (Hildebrandt et al., 2023). Orthopedic conditions, including osteoporosis and osteoarthritis, can trigger peripheral inflammatory responses. During the chronic pathological stages of osteoporosis, pro-inflammatory cytokines can stimulate the activation and differentiation of osteoclasts and upregulate the expression of receptor activator of nuclear factor κB ligand (RANKL), thereby reducing osteoblast survival and promoting bone resorption (Arra et al., 2020; Chang et al., 2009; De Roover et al., 2023). In the context of AD, the impairment of the BBB and its increased permeability allow peripheral inflammatory cytokines to enter the brain. This induces neuroinflammatory responses, neuronal damage, and neurodegenerative changes, effectively accelerating the progression of AD (Sousa et al., 2023).

### 3.3 Molecular pathways

Molecular pathways are sophisticated systems that precisely regulate cell growth, differentiation, and metabolism. Their aberrant activation or inhibition serves as a fundamental driver in the pathogenesis of various diseases. Within the “bone-brain axis” framework, the specificity of these pathways is manifested in their roles as cross-organ regulatory nodes. These nodes facilitate the directional transport and bidirectional interaction of signaling molecules between bone tissue and the nervous system.

#### 3.3.1 RANK/RANKL/OPG pathway

The RANK/RANKL/OPG signaling pathway, acting as a critical cross-organ link, plays a central role in the occurrence and progression of cognitive decline and osteoporosis (Gao et al., 2025; Marcadet et al., 2022). Receptor Activator of Nuclear Factor-κB Ligand (RANKL) is primarily secreted by osteoblasts and osteocytes; by binding to its receptor RANK, it promotes osteoclastogenesis and enhances bone resorption capacity (Yue et al., 2022).

Conversely, osteoprotegerin (OPG), secreted by osteoblasts, acts as a decoy receptor that specifically binds to RANKL. This prevents RANKL from interacting with RANK, thereby inhibiting the formation of osteoclasts and the subsequent bone resorption process. Under physiological conditions, the dynamic balance between RANKL and OPG maintains skeletal homeostasis; however, when this balance is disrupted—characterized by upregulated RANKL expression and downregulated OPG—it leads to excessive osteoclast activation, resulting in bone loss and eventual development of osteoporosis (El-Masri et al., 2024; Marcadet et al., 2022).

Notably, RANKL also serves as a bone-brain messenger; it is expressed within the brain and participates in the regulation of neuronal function. In neuroinflammatory states, the RANK/RANKL interaction serves as a regulatory node that modulates microglial activation, thereby altering the neuronal microenvironment. Research indicates that within this microenvironment, the RANKL/RANK axis can promote the secretion of pro-inflammatory cytokines such as tumor necrosis factor-α (TNF-α) and interleukin-1β (IL-1β). This, in turn, induces aberrant β-amyloid (Aβ) production and disrupts synaptic connectivity, ultimately translating skeletal metabolic stress into cognitive impairment (Glasnović et al., 2020).

#### 3.3.2 Wnt signaling pathway

The Wnt signaling pathway comprises a family of proteins that play a pivotal role in maintaining embryonic development and adult tissue homeostasis. Based on whether signal transduction depends on β-catenin, this pathway is categorized into the canonical Wnt pathway (also known as the Wnt/β-catenin pathway) and the non-canonical Wnt pathway (Liu J. et al., 2022).

Research has demonstrated that Wnt signaling serves as a vital hub connecting bone metabolism and cognitive function (Huang et al., 2025; Liu J. et al., 2022; Marini et al., 2023). In the pathogenesis of osteoporosis, various factors can disrupt normal bone metabolism by interfering with the Wnt signaling pathway. For example, bioactive oxidized phospholipids (oxPLs) can bind with LRP6 on the surface of bone marrow mesenchymal stem cells (BMSCs), inducing its internalization and inhibiting Wnt signaling. This reduces the differentiation capacity of osteoblasts, ultimately leading to decreased bone formation (Liu J. et al., 2022).

Similarly, the Wnt signaling pathway plays a crucial regulatory role in the pathological progression of Alzheimer’s disease (AD) (Elliott et al., 2018). Specifically, activation of the canonical Wnt pathway can inhibit AD pathology, whereas activation of the non-canonical pathway may promote disease development. Consequently, a decline in the functionality of the canonical Wnt pathway significantly promotes β-amyloid (Aβ) deposition, hyperphosphorylation of microtubule-associated protein tau, blood-brain barrier (BBB) disruption, and synaptic loss, thereby accelerating the onset and progression of AD (Wang Q. et al., 2022).

The cross-organ regulatory characteristics of Wnt signaling are explicitly manifested through its specific inhibitors, most notably Sclerostin (SOST) and Dickkopf-1 (DKK1). As potent inhibitors of the Wnt pathway, DKK1 is concurrently involved in the pathological regulation of both osteoporosis and AD (Kang et al., 2023; Wang Q. et al., 2022). In osteoporosis, DKK1 inhibits osteoblast function and reduces bone formation; meanwhile, in AD, it further promotes Aβ deposition and tau protein phosphorylation by blocking Wnt signaling. Research has revealed that serum DKK1 levels are significantly elevated in AD patients and positively correlate with cognitive decline. Simultaneously, DKK1 is recognized as a risk biomarker for osteoporosis. These findings further substantiate the central role of Wnt signaling in the cross-organ regulation of the bone-brain axis (Elliott et al., 2025; Simic et al., 2023).

#### 3.3.3 Notch signaling pathway

The Notch signaling pathway plays a fundamental role in cell fate determination and the maintenance of systemic homeostasis (Dilawar et al., 2025). Within the skeletal system, functional abnormalities in this pathway inhibit bone formation, leading to a decrease in bone mineral density (BMD) (Perepletchikova and Malashicheva, 2025; Xu et al., 2022). Within the regulatory framework of the bone-brain axis, the specificity of this pathway is centered on γ-secretase, which acts as a dual-functional regulatory node. γ-secretase is concurrently involved in both the activation of Notch receptors within bone metabolism and the processing of amyloid precursor protein (APP) in the brain. Consequently, aberrant γ-secretase activity serves as a molecular convergence point that not only interferes with Notch signal transduction in bone tissue but also alters the production of β-amyloid (Aβ), thereby accelerating the overall pathological progression of Alzheimer’s disease (AD) (Zhou et al., 2022).

## 4 Imaging methods

Cognitive decline and reduced bone mineral density represent two prevalent age-related health concerns, and a significant association has been established between them (Xie et al., 2023). Given the sustained global rise in cases of both dementia and osteoporosis, the determination of precise imaging diagnostic methods has become paramount (Nasiri et al., 2025).

### 4.1 Cognitive decline

**FIGURE 2:**
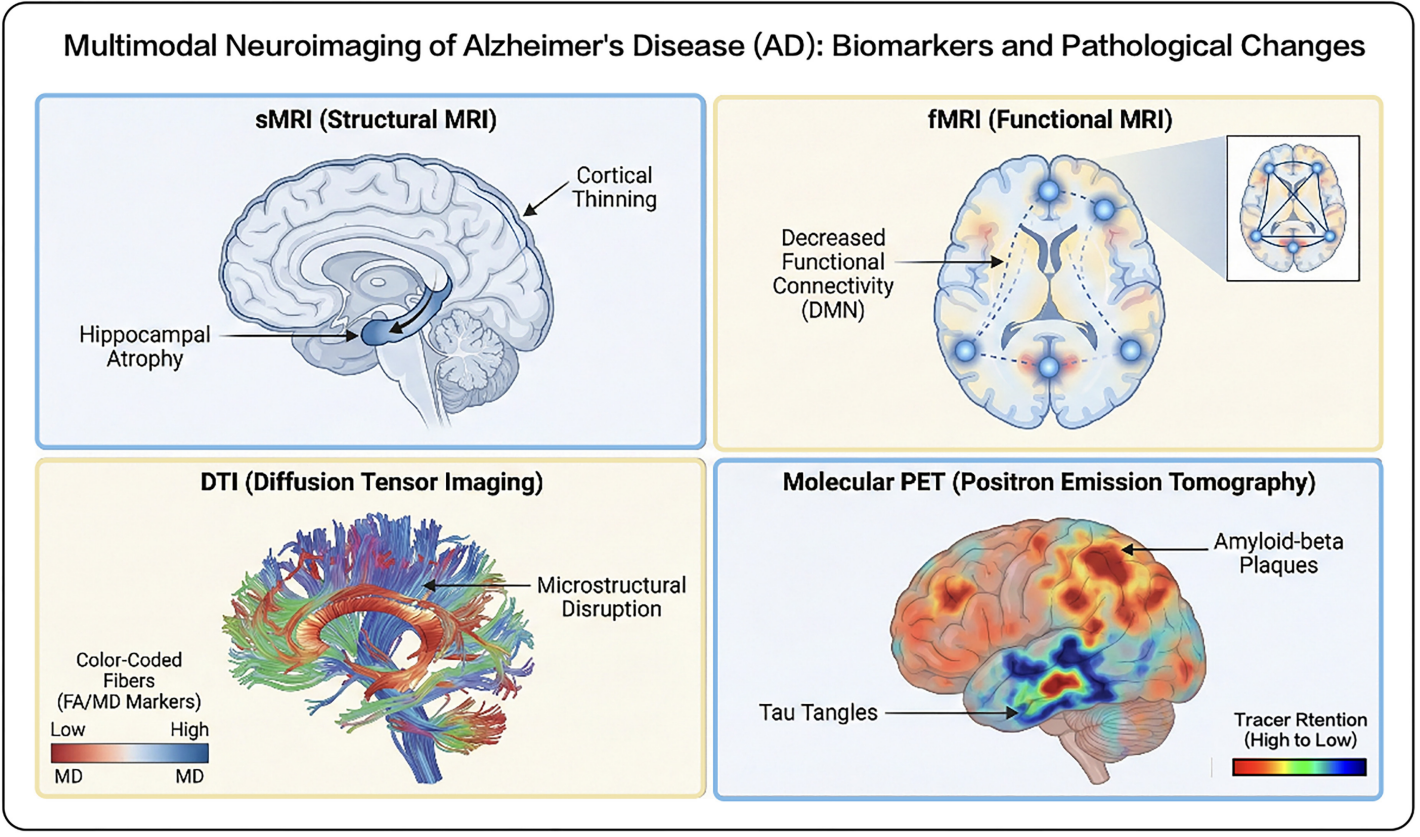
Overview of multimodal imaging biomarkers and pathological alterations across the Alzheimer’s disease continuum.

Neurodegenerative diseases are frequently characterized by pathological alterations, including brain regional atrophy, white matter damage, and aberrant functional activities. Imaging technology, particularly MRI, offers unique diagnostic value in detecting these macroscopic structural and functional lesions (Figure 2). Diverse MRI-derived imaging modalities have emerged as indispensable instruments for characterizing structural and functional connectivity while elucidating the pathogenic mechanisms underlying neurological disorders (Table 1; Gatto et al., 2022).

**TABLE 1:** Primary imaging modalities, indicators, and clinical correlations across the Alzheimer’s disease (AD) continuum.

Imaging dimension	Technical principles	Key quantitative indicators	Abnormal presentations in the AD continuum	Clinical significance and cognitive correlation
Structural MRI (sMRI)	Measures proton density and relaxation times (T1/T2) to generate high-resolution anatomical images.	Gray matter volume, cortical thickness, hippocampal volume.	Early detection of medial temporal lobe (hippocampus) and frontoparietal cortical thinning; significant whole-brain atrophy in middle-to-late stages (Choi et al., 2022; Gonuguntla et al., 2022; Horie et al., 2025).	Structural changes typically precede symptoms; hippocampal atrophy directly reflects the severity of memory impairment.
Functional MRI (fMRI)	Based on the “Blood Oxygen Level Dependent (BOLD)” effect, reflecting neuronal activity intensity (Wang M. et al., 2022).	Resting-state functional connectivity (rsFC), Default Mode Network (DMN) connectivity.	Reduced connectivity between the hippocampus and key DMN nodes (e.g., medial prefrontal cortex); disintegration of inter-network coordination (Haseltine et al., 2021; Link and Kazakia, 2020).	Captures early functional disturbances in neuronal activity; reflects overall dysregulation of cognitive and executive functions.
Diffusion tensor imaging (DTI)	Measures the direction and intensity of water molecule diffusion within white matter fiber tracts (van den Bergh et al., 2021).	Fractional anisotropy (FA), mean diffusivity (MD).	White matter microstructural damage manifests as decreased FA and increased MD; abnormal indicators in key pathways like the fornix hold differential diagnostic value (van den Bergh et al., 2021).	Assesses axonal integrity and myelination; serves as a sensitive means to detect microstructural changes during pathological progression (Li et al., 2024; Mesinovic et al., 2025).
Molecular PET imaging	Utilizes radiotracers to bind with specific protein targets to detect *in vivo* pathological protein distribution.	1. Aβ PET: Amyloid plaque deposition. 2. Tau PET: Density and distribution of neurofibrillary tangles (NFTs).	Early Aβ deposition in the cingulate gyrus and precuneus; Tau accumulation in the medial temporal lobe. Pathological spread to the neocortex in middle-to-late stages (Liu J. et al., 2022; Nakajima et al., 2025; Zhang et al., 2024).	Enables *in vivo* pathological visualization; elevated Tau levels are identified as the primary drivers of cognitive decline.

The neuropathological hallmarks of Alzheimer’s disease (AD) involve the deposition of extracellular amyloid plaques—composed of amyloid-β (Aβ) peptide aggregates—and intraneuronal neurofibrillary tangles (NFTs) resulting from the accumulation of hyperphosphorylated tau protein (Hyman et al., 2012; Montine et al., 2012). As a well-established molecular imaging technique, Positron Emission Tomography (PET) enables the *in vivo* detection and localization of these pathological protein aggregates (Rowe et al., 2017; Wolk et al., 2012). This is achieved by measuring the biodistribution of radiotracers that bind selectively and specifically to their respective protein targets (Leithner et al., 2024).

To facilitate the precise identification of patients in the early stages of AD, researchers have proposed the Amyloid (A) – Tau (T) – Neurodegeneration (N) framework (Jack et al., 2018). This framework is particularly efficacious for individuals in the preclinical stage who have not yet manifested clinical symptoms. Within this paradigm:

- “A” is assessed via imaging modalities such as Aβ PET to identify the distribution of amyloid plaques within the brain.
- “T” utilizes imaging techniques like tau PET to localize regions of neurofibrillary tangles.
- “N” employs structural imaging (e.g., MRI) to evaluate volume changes in specific brain regions, such as hippocampal atrophy (Choi et al., 2022; Duong et al., 2022; Gonuguntla et al., 2022; Horie et al., 2025).

### 4.2 Osteoporosis

**FIGURE 3:**
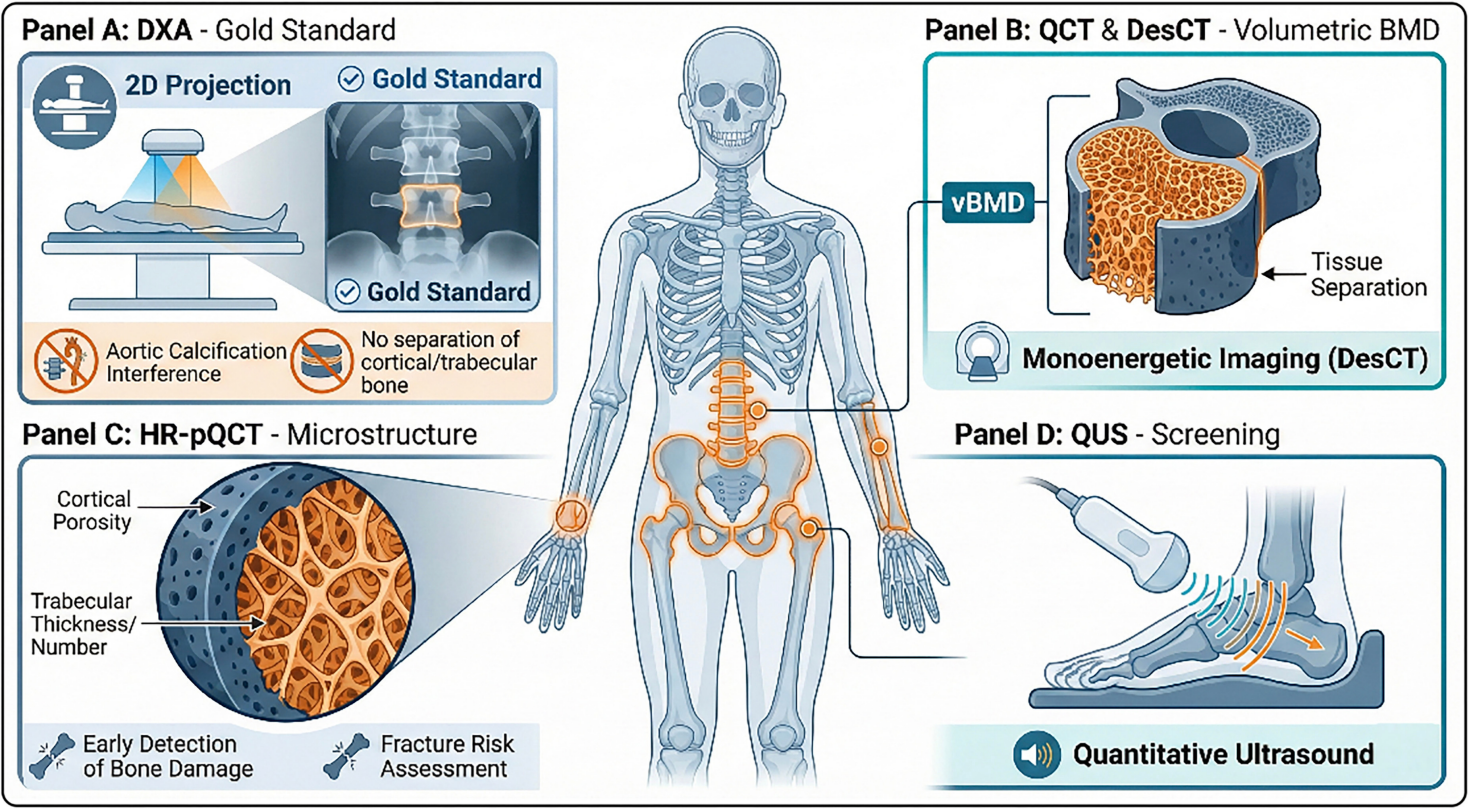
Overview of cutting-edge imaging modalities for the assessment of bone mineral density and microarchitecture. **(Panel A)** DXA; **(Panel B)** QCT & DesCT; **(Panel C)** HR-pQCT; **(Panel D)** QUS.

Currently, the primary imaging modalities for bone mineral density (BMD) measurement include Quantitative Ultrasound (QUS), Dual-Energy X-ray Absorptiometry (DXA), Dual-Energy Spectral CT (DesCT), Quantitative Computed Tomography (QCT), and High-Resolution Peripheral Quantitative Computed Tomography (HR-pQCT) (Haseltine et al., 2021; Link and Kazakia, 2020; Wang M. et al., 2022; Figure 3).

#### 4.2.1 DXA: the clinical “gold standard”

DXA is globally recognized as the current “gold standard” for BMD measurement, predominantly utilized for evaluating core skeletal sites such as the lumbar spine, hip, and forearm. This imaging technique offers advantages such as low radiation dosage and high measurement precision. However, it is constrained by its two-dimensional projection imaging principle; anatomical abnormalities—such as degenerative spinal changes or aortic calcification—can lead to an overestimation of BMD values. More crucially, DXA cannot achieve the compartmentalized detection of cortical and trabecular bone density.

#### 4.2.2 Advanced CT imaging: DesCT and QCT

- DesCT: By leveraging pseudo-monochromatic imaging technology, DesCT reduces X-ray beam hardening effects. Combined with bone tissue separation algorithms, it enables quantitative BMD measurement. Research data indicates that DesCT-derived BMD values exhibit excellent stability and repeatability across varying radiation doses and correlate highly with QCT results, suggesting significant clinical potential for spinal bone density assessment.
- QCT: QCT allows for true volumetric BMD measurement of the lumbar spine and proximal femur. Its core advantage lies in the fact that results are unaffected by body size, and it can precisely distinguish between cortical and trabecular bone for compartmentalized quantification. Nevertheless, the relatively high radiation dose associated with QCT somewhat limits its widespread application in routine screening and long-term follow-up.

#### 4.2.3 HR-pQCT: microstructural excellence

High-resolution peripheral quantitative computed tomography is a specialized imaging technology for the microstructural evaluation of peripheral bone. It enables the precise quantification of cortical porosity and the simultaneous assessment of multiple microstructural parameters, including trabecular thickness, number, and separation. This capability addresses the deficiencies of traditional imaging methods that focus solely on density while neglecting microstructural integrity.

In the progression of conditions such as osteoporosis and diabetes-related bone disease, increases in cortical porosity typically precede declines in BMD. By detecting this sensitive indicator, HR-pQCT facilitates the early identification of bone quality impairment (van den Bergh et al., 2021). Furthermore, the cortical porosity changes detected by HR-pQCT can be correlated with bone strength indicators (such as stiffness and failure load). This not only aids in the early detection of bone quality damage but also provides a more comprehensive reference for fracture risk assessment, further expanding its clinical utility in metabolic bone diseases (Mesinovic et al., 2025).

### 4.3 Bone-brain multimodal imaging fusion

Within the theoretical framework of the regulatory mechanism underlying the bone-brain axis, there exist close physiological synergistic effects and pathological interactions between brain tissue and bone tissue (Li et al., 2024). Although a single imaging modality can provide basic baseline data, it is insufficient to fully elucidate the complex bidirectional regulatory characteristics of the bone-brain axis. In-depth exploration therefore relies on an integrated and multi-scale imaging research system. Multi-scale matched detection enables more sensitive identification of synergistic degenerative pathological changes in the bone and brain. Existing multimodal studies have yielded solid evidence: atrophy of motor-related brain regions is bidirectionally correlated with decreased lumbar bone mineral density (BMD) (Zhang et al., 2024); in patients with diabetic osteoporosis (DOP), enhanced spontaneous neural activity in brain regions such as the left middle temporal gyrus—with regional homogeneity (ReHo) as the core indicator—shows a significant positive correlation with bone mineral density (Liu M. et al., 2022); osteoporosis is also closely associated with gray matter volume reduction in the right hippocampus, amygdala, and parhippocampal gyrus (Nakajima et al., 2025). In summary, conducting *in vivo* multimodal research by integrating cranial imaging techniques with bone mineral density detection protocols constitutes a core approach to revealing the intrinsic mechanisms of bidirectional bone-brain regulation. Furthermore, the synchronous evolutionary patterns across multiple indicators and modalities provide robust imaging support and objective evidence for the construction of combined clinical prediction models (Table 2).

**TABLE 2:** Integrated multimodal imaging research framework combining cranial imaging features with bone mineral density data to elucidate the synergistic pathological progression of the bone-brain axis and provide support for the construction of combined clinical risk prediction models.

Evaluation objective	Technical combination	Integrated advantages	Special value for bone-brain axis research
Early risk prediction	HR-pQCT + sMRI	The sensitivity of HR-pQCT to bone microarchitecture complements that of sMRI to hippocampal atrophy.	Enables establishment of a combined “bone microarchitecture–brain structure” predictive model before clinical symptoms appear.
Pathological mechanism linkage	Dual-tracer PET + QCT	Simultaneously quantifies bone metabolic activity and cerebral protein deposition, directly validating central effects of bone-derived factors.	Addresses the bottleneck that a single indicator cannot clarify the causal relationship between bone loss and protein deposition.
Clinical large-scale screening	DXA + neuropsychological scales	Low cost and low radiation exposure, suitable for large-sample epidemiological studies.	Establishes a baseline risk spectrum of bone-brain axis impairment to identify high-risk populations.
Network connectivity analysis	fMRI + DTI + QCT	Evaluates the physical connectivity of the bone-brain axis from both functional and white matter integrity dimensions.	Reveals the hierarchical pathway of “bone-derived signals–network disruption–cognitive decline.”

### 4.4 Technological innovations and future directions

Compared to traditional imaging technologies, dual-tracer PET offers unique advantages. By utilizing two specific probes to simultaneously target different molecular markers in the brain, it can capture the association between critical pathological processes—such as neuroinflammation, synaptic dysfunction, and amyloid deposition—and bone metabolism disturbances. This compensates for the limitations of single-modality and traditional multimodal techniques at the molecular level (Blake et al., 2018; Fu et al., 2014).

Building on this foundation, deep learning models (such as 3D-CNN and Graph Neural Networks) can construct joint prediction models through cross-modal feature extraction and transfer learning. This enables a transition from single biomarkers to multi-dimensional risk profiles, effectively enhancing the accuracy and sensitivity of screening.

However, key scientific and technical bottlenecks remain, primarily in how to effectively translate the theoretical results of integrated BMD and brain imaging analysis into clinically applicable diagnostic and therapeutic strategies. While some relationships between bone density and brain health have been revealed, sufficient clinical evidence is still lacking (Guo et al., 2023). Future research must incorporate large-scale cohorts across different age groups, genetic backgrounds, and health statuses. Long-term longitudinal follow-up and dynamic monitoring of the evolution between BMD and brain imaging indicators are essential to establish their correlation with the risk of Alzheimer’s disease (AD). Furthermore, translating molecular mechanism research into imaging-based and AI-driven early diagnostic tools and risk prediction models will provide the technical support and empirical basis for the early precision diagnosis of AD.

## 5 Conclusion

In the context of the accelerating global aging process, cognitive impairment and osteoporosis have emerged as serious diseases threatening the physical and mental health of the elderly. This review systematically elucidates that the two conditions are closely linked through the complex bidirectional signaling network of the “bone-brain axis” across epidemiological, pathophysiological, and molecular dimensions.

At the clinical evaluation level, single-modality imaging struggles to fully capture the complex bidirectional associations between bone and brain. Integrating brain structural, functional, and molecular imaging with bone mineral density (BMD) measurement data provides a unique perspective for analyzing bone-brain interactions *in vivo*. Dual-tracer PET technology, by targeting different molecular markers in the brain simultaneously, can capture the associations between neuroinflammation, synaptic dysfunction, AD-related protein deposition, and bone metabolism disturbances, offering critical technical support for interpreting “bone-brain” molecular mechanisms. Furthermore, combining deep learning models such as 3D-CNN and Graph Neural Networks to extract and analyze cross-modal imaging features allows for the construction of highly sensitive and accurate comorbidity risk prediction models. This facilitates a leap from single biomarkers to multi-dimensional risk profiles, providing essential support for early identification and intervention in high-risk populations.

At present, research on the regulatory mechanisms of the bone–brain axis faces prevalent common limitations: small sample sizes, a lack of large cohort studies covering different age stratifications, genetic backgrounds and health statuses, and insufficient long-term longitudinal follow-up data, which hinders comprehensive analysis of the dynamic evolutionary patterns between bone mineral density and neuroimaging indicators as well as their correlations with Alzheimer’s disease risk. Additionally, the absence of unified and standardized multimodal evaluation protocols, coupled with substantial heterogeneity in imaging techniques and analytical methods, results in poor comparability and reproducibility of research findings. Furthermore, this field encounters unique cross-organ research challenges and technical bottlenecks. Most existing studies adopt a cross-sectional design, making it difficult to clarify the causal relationships and chronological sequence of pathological alterations—this critical shortcoming severely restricts mechanistic interpretation, causal verification and clinical translation. The distinctive cross-organ difficulties are reflected in the following aspects. First is the contradiction of multi-scale biological asynchrony: bone remodeling occurs on a monthly timescale, while neural activity operates on a second-by-second basis. Such striking differences in temporal scales increase the difficulty of longitudinal imaging data alignment and causal inference. Second is the barrier to integrating heterogeneous characteristics: effectively mapping microstructural damage in mineralized bone detected by HR-pQCT to macroscopic functional brain networks captured by fMRI remains a major computational obstacle for AI-driven risk prediction models. The interplay of these general shortcomings and unique challenges constitutes the core barrier to in-depth research on the regulatory mechanisms underlying the bone–brain axis.

**FIGURE 4:**
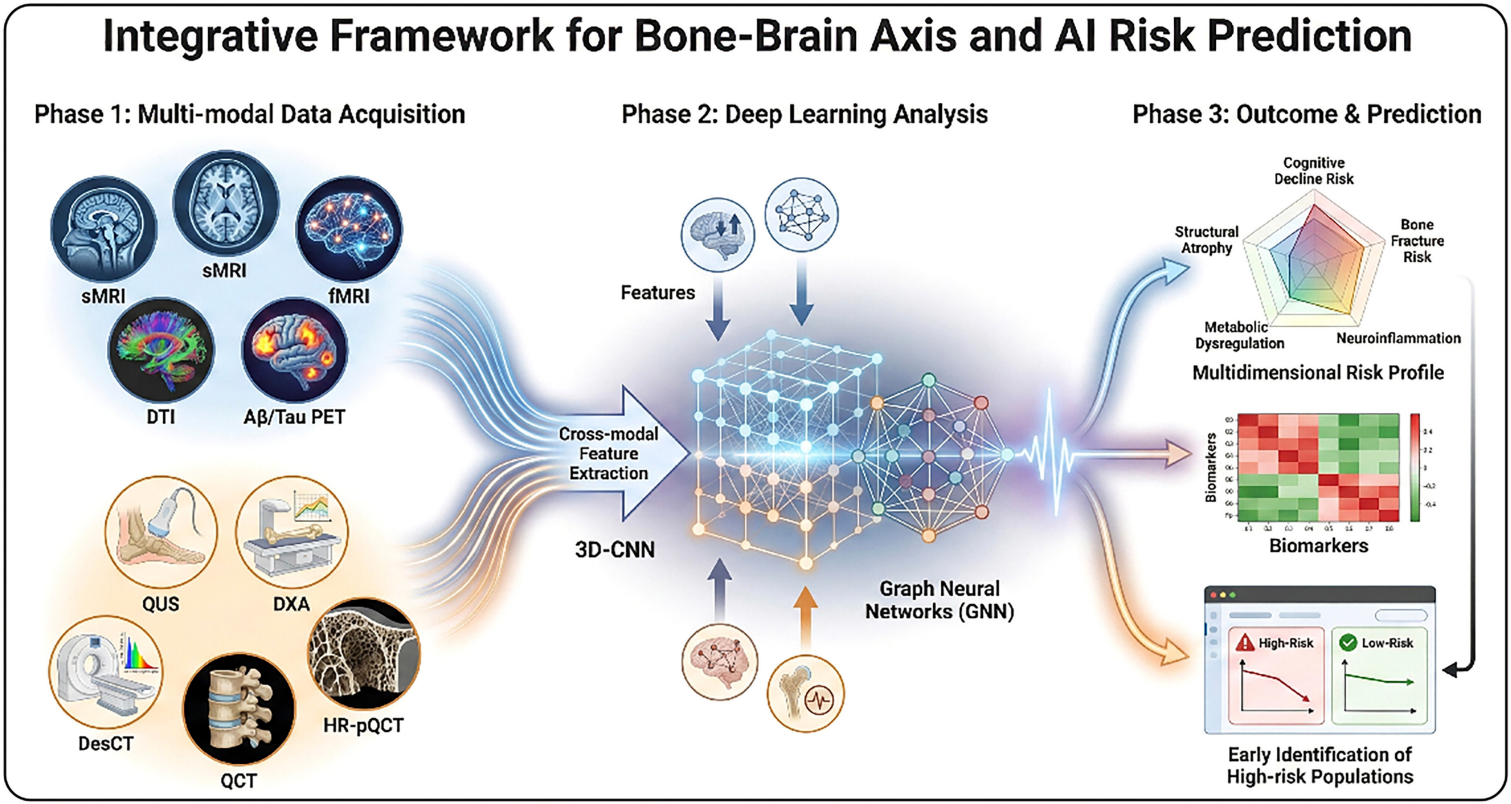
An integrated framework for cross-modal data fusion and AI-driven risk prediction based on the “bone-brain axis” theory.

Future research should focus on these key issues by conducting large-scale, prospective longitudinal studies with diverse populations to clarify causal relationships and temporal characteristics. Standardizing multimodal screening tools and unifying detection workflows will enhance the reliability of findings. Advancing translational medicine by combining AD molecular findings with multimodal imaging and AI models will enable the development of efficient early diagnostic tools and risk prediction models, eventually validated through large-scale clinical research (Figure 4).
